# How Long Do Older Adults Remain Homebound in the Community? Implications for Long-Term Services and Support Systems

**DOI:** 10.1093/geroni/igaa057.2504

**Published:** 2020-12-16

**Authors:** Katherine Ornstein, Jennifer Reckrey, Evan Bollens-Lund, Katelyn Ferreira, Mohammed Husain, Shelley Liu, Albert Siu

**Affiliations:** Icahn School of Medicine at Mount Sinai, New York, New York, United States

## Abstract

A large and growing population of older adults with multimorbidity, cognitive impairment, and functional disability live in the community but are homebound (never/rarely leave home). While homebound status is associated with decreased access to medical services and poor health outcomes, it is unclear how long individuals remain homebound. We used the National Health and Aging Trends Study (NHATS), a nationally representative sample of Medicare beneficiaries age 65 and over, with survey weighting to assess duration of homebound status in the community. Among the incident homebound in 2016 (n=253) , only 28% remained homebound after 1 year. 21% died, 18% were recovered, and one-third left the home but still reported difficulty. As the locus of long-term care shifts from nursing homes to the community and models of care expand to serve the needs of the homebound, it is critical that we better understand the heterogeneity and transitions of the homebound population.

